# Focus on One Swimming Stroke or Compete in Multiple: How Much Specialization Is Needed to Become a World-Class Female Swimmer?

**DOI:** 10.3390/jfmk10010064

**Published:** 2025-02-13

**Authors:** Dennis-Peter Born, Jenny Lorentzen, Jesús J. Ruiz-Navarro, Thomas Stöggl, Michael Romann, Glenn Björklund

**Affiliations:** 1 Swiss Development Hub for Strength and Conditioning in Swimming, Swiss Swimming Federation, 3048 Worblaufen, Switzerland; 2 Department for Elite Sport, Swiss Federal Institute of Sport Magglingen, 2532 Magglingen, Switzerland; 3 Faculty of Science and Medicine, University of Fribourg, 1700 Fribourg, Switzerland; 4 Aquatics Lab, Department of Physical Education and Sports, Faculty of Sport Sciences, University of Granada, 18071 Granada, Spain; 5 Red Bull Athlete Performance Center, 5303 Thalgau, Austria; 6 Swedish Winter Sports Research Centre, Mid Sweden University, 831 40 Östersund, Sweden

**Keywords:** competitive swimming, deliberate practice, diversification, long-term athlete development, sampling

## Abstract

**Objectives:** To investigate performance development and variety in swimming strokes of female swimmers from early junior to elite age. **Methods**: A total of 194,788 race times of female 200 m swimmers representing 77 nations were ranked at peak performance age and clustered into world-class finalists (>850 swimming points), international-class (750–850), national-class (650–750) and regional-class swimmers (550–650). Annual best times for each swimming stroke were retrospectively extracted throughout adolescence from 13 years of age. Longitudinal performance development and differences between the swimmers’ main and their secondary swimming strokes were analyzed using linear mixed model. **Results**: World-class freestyle swimmers show significantly (*p* ≤ 0.042) higher swimming points across all age categories compared to international-, national- and regional-class swimmers. Linear mixed model analysis indicates a significant performance progression for international- and national-class freestyle swimmers up to the 19–20-year-old category (*p* ≤ 0.038), but an earlier plateau was observed for regional-class swimmers (*p* = 0.714). Comparing main and secondary swimming strokes, freestyle swimmers show the highest degree of specialization. For breaststroke and individual medleys, specialization increases with increasing performance level and the closer an athlete is to elite age. World-class butterfly and backstroke finalists show the lowest specializations in terms of the smallest number of significant differences compared to performances in their secondary swimming strokes. **Conclusions**: Higher ranked swimmers show a greater degree of specialization. As different specialization patterns are evident for the various swimming strokes, decision makers and talent specialists should align development guidelines accordingly and base them on the most advantageous combinations of swimming strokes.

## 1. Introduction

Should athletes specialize during their early career or maintain a large variety of technical skills and physical conditioning? This topic is controversially and heavily debated in the field of sport science and talent development [1,2,3,4,5,6,7]. As such, some studies found that world-class athletes generally enter their main sport later, have less specific training and are instead more involved in other sports compared to their lower ranked peers [1]. Additionally, it has been discussed that there may be an inverse relationship between involvement in talent development programs and success as an adult athlete [2]. On the other hand, and specifically for swimming, Yustres and co-workers [4] show that success at junior and senior World Championships are related, in particular for female swimmers [5]. Mainly, participating in junior World Championships across multiple years increases success chances at adult age [6]. Therefore, successful elite age swimmers begin to show superior performances compared to their lower ranked peers from the age of 12 years onward [7].

Confusion may result from the yet missing, generally accepted definition of the term “sport specialization” [8,9], which for the present investigation refers to the “intentional and focused participation in” particular swimming strokes [8], rather than diversification across butterfly (BU), backstroke (BA), breaststroke (BR), freestyle (FR) and individual medley (IM). Caution should be exercise when generalizing the effects of involvement in a variety of sports versus specialization patterns across various disciplines of a particular sport, especially when mixing results from various methodological approaches. While some studies used retrospective questionnaires—which rely on the participants’ accurate memory—for the assessment of sport involvement [10,11], recent studies have used race results as an objective measure to quantify specialization patterns within the specific sports [3,5,7,12]. This is particularly feasible for swimming, as competitions involve highly standardized conditions in regard to pool size, water temperature, current and wave breaking lanes [13]. However, previous studies mainly assessed the relationships between success of swimmers as both juniors and adults [3,5,7]. Little attention has been paid to within-sport specialization patterns, i.e., the involvement in various swimming disciplines and performance differences between the main and secondary swimming strokes.

A previous study quantified variety based on the number of different swimming strokes, and the results show a dose–time effect. As such, FR swimmers benefit from a large variety of swimming strokes as juniors and a stepwise specialization to two swimming strokes as adults [14]. However, specialization patterns are specific for each swimming stroke. The assessment of performance differences between the swimmers’ main and secondary swimming strokes shows that world-class BR finalists specialized earlier and more, while world-class FR and IM finalists specialized less compared to their lower ranked peers [15]. However, this study only involved male swimmers. Due to earlier maturation [16], female swimmers have to perform at a higher level (closer to the world record) at a younger age than their male counterparts [17], which may alter the specialization pattern and require a sex-specific analysis.

As there are ever more demands for well-balanced research on both male and female athletes from journal policies and editorials [18,19,20], the present study focuses on specialization patterns in female swimmers and aims to (1) investigate performance development in a representative group of competitive female swimmers from early junior to elite age across a large range of performance levels (world-class finalists vs. international-, national- and regional-class swimmers) and to (2) assess variety by comparing the swimmers’ main swimming strokes with their secondary ones. Since specialization patterns are expected to be different between the various disciplines, the data analysis was conducted independently for all swimming strokes.

## 2. Materials and Methods

### 2.1. Subjects

A total of 194,788 race times of 1464 female swimmers representing 77 nations and competing at peak performance age were provided by the official database (Swimranking, Splash Software Ltd., Spiegel b. Bern, Switzerland) of the European Swimming Association LEN (Ligue Européenne de Natation) with permission for scientific analysis and the publication of the results. Since only publicly available data were utilized, formal written consent from the swimmers was not necessary. The host of the database provided the data completely anonymized. No subject could be identified before, during or after the data analysis. The study protocol was approved in advance by the institutional review board of the Swiss Federal Institute of Sport Magglingen (Reg.-Nr. 177_LSP_102022, approval data: 25 October 2022) and adhered to the ethical standards of the World Medical Association concerning research involving human subjects, which are outlined in the Declaration of Helsinki.

### 2.2. Data Analysis

The data of female swimmers who competed in the 200 m events at peak performance age during the recent Olympic cycle (2016 until 2021) were extracted from the database. Peak performance ages have previously been determined for top elite female 200 m BU (22 years), BA (22 years), BR (24 years), FR (22 years) and IM (22 years) swimmers [21]. The 200 m long-course events (50 m pool length) were chosen for this initial step of the analysis, as this is the only Olympic race distance that represents all swimming strokes. Swimming points were calculated based on the official method of the world governing body of swimming (World Aquatics) to express race times relative to the current world record [13] and to compare performances between the various swimming strokes and race distances. The swimming points of the personal best race times in specific 200 m events during peak performance age (as mentioned above) were used to rank the swimmers. Subsequently, annual best race times were retrospectively extracted from peak performance age throughout adolescence from 13 years of age for all swimmers. To encompass the entirety of the developmental process, all race distances, short-course (25 m pool length) and long-course races were accounted for during this second step of the analysis. Short-course world records were used to calculate swimming points for short-course races.

To compare swimmers of different performance levels and investigate their longitudinal development, swimmers were clustered into performance levels and age categories. Performance levels were defined based on swimming points at peak performance age: swimmers who reached more than 850 swimming points (850) were classed as world-class finalists, while those who reached 850–750 swimming points (750), 750–650 swimming points (650) or 650–550 swimming points (550) were classed as international-, national- or regional-class swimmers, respectively [22]. Swimmers who scored fewer than 550 swimming points at peak performance age were excluded from any further analyses. Mean annual best times were computed for the following age categories: 13–14, 15–16, 17–18, 19–20 and 21+ (21–26) years. Although specific ages (22, 22, 24, 22 and 22 years for female 200 m BU, BA, BR, FR and IM swimmers, respectively [21]) were used to rank the swimmers based on their personal best at peak performance age in the initial step of the analysis, one common age category (21–26 years) was used for the retrospective analysis and to allow the comparison of performance development and specialization patterns between the various swimming strokes. Potential deviations between the statistically determined peak performance age for top elite swimmers [21] and the actual age at which the personal best across all race distances within the particular swimming stroke occurred are presented in the Supplementary Materials (Table S1).

The ‘pandas’ library (version 1.5.1, pandas-dev/pandas, Zenodo, Genève, Switzerland) for Python (version 3.9.7, Python Software Foundation, Beaverton, OR, USA) was utilized to calculate swimming points, establish rankings at peak performance age and conduct the retrospective analysis of annual best race times throughout the swimmers’ careers. Subsequent data processing was carried out using Microsoft Excel (version 2209, Microsoft Corporation, Redmond, WA, USA).

### 2.3. Statistical Analysis

All data are presented as means ± standard deviation. Normality was confirmed with standardized residuals displayed as a diagonal straight line in the Q-Q plot and a Gaussian distribution in the histogram [23]. Non-normally distributed data were logarithmically transformed. *Performance levels* (850 vs. 750 vs. 650 vs. 550 swimming points) across the *age categories* (13–14 vs. 15–16 vs. 17–18 vs. 19–20 vs. 21+) were compared by linear mixed model analysis with fixed intercepts and restricted maximum likelihood. Swimming points during each age category were used as the dependent variables, while *performance levels* and *age categories* were used as fixed factors and the *subject ID* as the random factor. Bonferroni’s correction was applied to the post hoc tests for the pairwise comparison. An alpha level of 0.05 was set for all analyses, which were performed with the Jamovi software package version 2.3.28.0 (Jamovi Project 2022, retrieved from https://www.jamovi.org).

## 3. Results

### 3.1. Performance Development

World-class FR swimmers show significantly higher swimming points across all age categories, i.e., 13–14 (*p* ≤ 0.042), 15–16 (*p* < 0.001), 17–18 (*p* ≤ 0.013), 19–20 (*p* < 0.001) and 21+ years (*p* < 0.001), compared to international-, national- and regional-class swimmers. For the other swimming strokes, i.e., BU, BA, BR and IM, world-class finalists do not significantly differ from international-class swimmers (except for during the 13–14 age category in BR). For these swimming strokes, significantly lower swimming points are only evident for national- and regional-class swimmers (*p* < 0.05). The difference in mean swimming points between world-class finalists and international-class swimmers increases from early junior (13–14 years) to elite age (21+ years) in FR (+31), BA (+38) and IM (+37) swimmers, but not in BU (+1) or BR (−4) athletes. The linear mixed model analysis shows a significant performance development of international- and national-class FR swimmers until the 19–20 age category (*p* ≤ 0.038). The performance of regional-class swimmers plateaus earlier during the 17–18 age category (*p* = 0.714). The trend analysis of mean swimming points shows the continuously improving performance development of world-class finalists and international-class swimmers, even between the last two age categories (19–20 and 21+ years), i.e., FR: +14 and +9; BU: +7 and +21; BA: +29 and +21; BR: +42 and +26; and IM: +21 and +20. In contrast, performance progression plateaus and even declines for national- and regional-class swimmers in FR (−1 and −10), BU (−2 and −11), BA (+6 and −6) and IM (+2 and −8), respectively (Table 1).

### 3.2. Specialization Patterns

**Figure 1:**
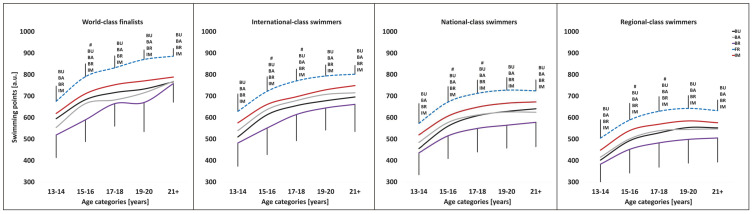
Performance progression of **freestyle** (FR) swimmers across the various age categories and performance levels. ^BU^ (butterfly), ^BA^ (backstroke), ^BR^ (breaststroke) and ^IM^ (individual medley) within the graphs indicate the significant differences between the main (dotted line) and the other swimming strokes. For the sake of clarity, significant differences to the previous age category (#) are only indicated for the main swimming stroke.

Figure 1 shows the specialization patterns in regard to the performance differences between the main swimming stroke (dotted line) and the secondary ones. The figure illustrates the performance progression for the various performance levels, i.e., world-class finalists and international-, national- and regional-class swimmers. FR swimmers of all performance levels and age categories show the highest degree of specialization, with significantly faster FR than BU, BA, BR and IM performances. In contrast, in BR and IM, the specialization depends on performance level, with higher performing swimmers showing greater specialization and world-class BR and IM finalists continuously increasing their specialization as they near elite age. World-class BU and BA finalists show the lowest degree of specialization with the least number of significant differences their performances in the main compared to the secondary swimming strokes (Appendix A, Appendix B, Appendix C and Appendix D).

In the upper performance levels, i.e., 850, 750 and 650 swimmers, the main swimming stroke (identified by ranking at peak performance age) always shows highest swimming points, while regional-class swimmers always showed fastest performances in FR, despite their appearance in the BU, BA or IM ranking at peak performance age. Only regional-class BR swimmers showed fastest performances in BR (refer to Appendix C).

When comparing performance of the oldest age category (21+), there are more significant differences between swimmers’ main and secondary swimming strokes the higher the performance level in BA, BR and IM. Additionally, the trend analysis reveals a greater performance gap between the swimmers’ main and other swimming strokes across all stroke types as performance level increases (850 vs. 750 vs. 650 vs. 550 swimmers). Performance trajectories (slope of annual performance progression) across the age categories (from early junior to elite age) are steeper at higher performance levels.

When comparing the combination of swimming strokes, BR is the slowest swimming stroke for swimmers with BU, BA and FR as their main stroke. This is evident across all performance levels from 850 to 550 swimmers. In contrast, BR is the second fastest swimming stroke at elite age for world-class IM swimmers and, vice versa, IM is the second fastest swimming stroke for world-class BR specialists. World-class BU and BA swimmers show fast FR and IM performances, but do not combine well with each other (refer to Appendix A and Appendix B, respectively).

## 4. Discussion

Swimming performance is significantly different between female world-class FR finalists and international-, national- and regional-class female FR swimmers across all age categories from early junior (13–14 years) to elite age (21+ years). While regional-class swimmers show an early performance plateau, performance trajectories (slope of annual improvements) are steeper the higher the performance levels, i.e., increasing annual progression from national-class swimmers to world-class finalists. Additionally, specialization increases from regional-class swimmers to world-class finalists. Specialization, based on significant differences between the main and the secondary swimming strokes, is greatest in world-class FR finalists across all age categories, followed by world-class BR and IM finalists, who show increased specialization throughout their age categories. World-class BU and BA finalists show the lowest specialization. Regarding stroke combinations, BR combines well with IM but not with BU, BA and FR. BU and BA do not combine well with each other but instead should be paired with FR and IM. Specialists across all swimming strokes also perform well in FR, which becomes particularly dominant in the lower performance levels.

The present study shows a significant difference between FR world-class finalists (850 swimmers) and their lower ranked counterparts. This was evident across all age categories, even during early junior age (13–14 years), which in contrast to the progression of male swimmers, who do not differ significantly before the 17–18 age category [15]. While male world-class finalists only have 507 ± 85 swimming points at the 13–14 age category [15], the present study shows that female world-class finalists of the same chronological age have a higher performance level and are at 684 ± 67 swimming points. Earlier biological maturation in women contributes to higher performance levels compared to men at the same chronological age and explains a proportion of the early separation of the performance groups [16]. Additionally, female swimmers generally have greater joint mobility [24] and have a more advantageous body composition for swimming than their male counterparts. Specifically, women have a lower center of mass and slightly higher body fat content even prior to puberty [25,26], as well as a body shape with better hydrodynamic characteristics [27]. As a result, the lower leg sinking torque and better buoyancy [28] may allow young female swimmers to focus on their propulsive skills, i.e., ‘catch’ at the beginning of the arm stroke, hydrodynamic lift and body rotation at a younger age. These technical elements, which contribute to optimal floating abilities and minimal drag forces, are important for swimming [29,30,31] and may result in the earlier separation of female talents from their less talented peers, thus allowing for earlier performance progression and earlier specialization. Interestingly, the early separation between the 850 and lower ranked swimmers was evident only for FR but not the other swimming strokes, which competitiveness and performance depth may account for.

Performance trajectories (slope of annual improvements) are steeper the higher the performance level, i.e., world-class finalists vs. the lower ranked swimmers. In contrast, regional-class swimmers show an early leveling-off in their development across the age categories, which is in line with previous research showing that both the initial performance level as well as progression are important contributors to top performance at elite age [32,33]. Interestingly, the present study shows differences in performance progression between the swimming strokes. As such, national- and international-class FR swimmers plateau at 21+ years, one age category later than the other swimming strokes (BU, BA, BR and IM). Different competitive levels and performance depths between the swimming strokes may account for these differences [34]. Additionally, the importance of the prestigious 4 × 200 m FR relay may encourage specialists of other swimming strokes to continue to progress in their 200 m FR performance.

Specialization increases as performance level increases, with the largest differences between main and secondary swimming strokes in world-class finalists. This is in line with previous research in male swimmers [15] and is probably related to the number of quality sessions required per week, i.e., speed and high-intensity work, that have to be performed in the specific movement pattern and velocity of the targeted event, respectively [35]. When comparing swimming strokes, world-class FR finalists show the highest degree of specialization. A total of six race distances at pool competitions allows FR swimmers to compete in multiple events with their main swimming stroke. Since “swimmers are stroke specialists rather than distance specialists” [36], swimmers can transfer their technical skills more easily to multiple race distances, rather than competing in multiple swimming strokes over a single race distance. However, BU and BA events are limited to a maximum of three race distances each, i.e., 50 m, 100 m and 200 m (even limited to two race distances each at the Olympic Games), and thus, world-class BU and BA finalists generally add a second swimming stroke to their competition schedule to increase their medal chances. This results in BU and BA specialists showing the largest variety across the five swimming strokes.

Interestingly, world-class BR finalists show a high degree of specialization, despite the same limitation to three race distances. This may be due to the technical characteristics of BR swimming. While the other swimming strokes share multiple technical similarities, such as undulating kicking applied during the underwater phase, the initial catch and high elbow position during the arm stroke (BU and FR), as well as the flutter kick (BA and FR) [37,38], the unique leg kick, large vertical displacement, high horizontal velocity changes and low movement economy of BR swimming seems to require a high degree of specialization [39,40]. The technical uniqueness may also explain why BR is the slowest swimming stroke for BU, BA and FR specialists. In contrast, BR is the second strongest stroke for world-class IM swimmers at peak performance age and, vice versa, IM the second-best stroke for BR swimmers. Naturally, none of the swimming strokes can be neglected when regarding the high competitiveness among world-class IM finalists. However, due to the slowest swimming velocity and largest timely contribution [41], BR is particularly important for IM races [42,43].

Interestingly, BU combines better with FR than with BA, despite it being a simultaneous (BU) rather than an alternating swimming strokes (FR) in regard to arm and leg movement. However, BU and FR are both performed in a prone body position and share common movement patterns for their arm stroke, in particular for the initial catch, the subsequent propulsive phase and high involvement of chest muscles [38,44,45]. Additionally, FR swimmers may benefit from the BU-specific undulating kicking for their underwater phase after starts and turns [37]. While BA may appear similar to FR, it requires swimmers to execute arm strokes at the side of the body, i.e., at biomechanically less efficient joint angles and with less involvement of the chest muscles [44]. Additionally, BA requires a larger shoulder roll with a decoupled hip rotation than FR, which impairs streamlining when not executed optimally [46,47]. It is noteworthy that specialists in various swimming strokes do well in FR. This is probably due to the fact that FR is commonly used by all stroke specialists for general warm-up, cool-down and long aerobic training sets.

However, one must distinguish between within- and between-sport specialization. While a broader movement foundation from the involvement in different sports during earlier junior age may aid performance at adult age [1,2,48,49,50], more successful athletes show a greater within-sport specialization, particularly during late junior age and the transition to adult age [15]. Based on the dose–time effect that shows successive specialization nearer to adult age [14], one may think of a specialization-pyramid, with a wide base comprising technical skills built as a junior athlete and slimming to a sharp tip (i.e., greater specialization) nearer adult age, allowing an athlete to be successful in specific events. This is supported by previous findings, suggesting that specialization develops along a continuum from early junior to senior age [51]. While “some degree of sports specialization is necessary to develop elite-level skill development” [51], premature or too much specialization increases the risk of overuse injuries [52]. Therefore, high degrees of specialization should not be undertaken before puberty [51], age-adequate free play should be allowed and functional conditioning programs should be encouraged to reduce overuse injuries [48,49]. It should be noted that modern swim training typically involves alternative activities and land-based exercises [35,53]. Such conditioning programs should incorporate preventive exercises in addition to resistance training aiming to improve neuro-muscular power. Future studies should account for those activities when investigating specialization patterns, since dry-land exercises potentially contribute to sport diversification, despite being part of a structured training program.

Particularly, for low-level (regional-class) swimmers, mean swimming points in the 19–20 and 21+ (21–26 years) age categories were higher than the thresholds (e.g., 550–650 for regional-class swimmers) used to classify swimmers’ performance levels (refer to Table 1). In the initial step of the analysis, swimmers were ranked based on their personal best 200 m race time of a particular swimming stroke at specific peak performance ages, determined by previous research (22, 22, 24, 22 and 22 years), for BU, BA, BR, FR and IM, respectively [21]. To cover the entity of an individual swimmer’s stroke-specific performance development, annual best times were retrospectively determined based on all race distances within the particular swimming stroke. Additionally, to allow the comparison of development patterns between the various swimming strokes, common age categories were formed for all events, i.e., the 21–26 age category that represents peak performance age. Since swimmers may show their personal best performances before or after the statistical mean for peak performance age, mean ± SD values for the broad 5-year age category (21+) indicate higher values than the specific thresholds of the performance levels. Since previous research determined peak performance age for top elite swimmers (top 16 in their respective events at the Olympic games) [21], discrepancy between the previously determined and the actual peak performance age of the present study was largest in the lowest performance level (regional-class swimmers; refer to Table S1). Hence, future studies and long-term athlete development strategies need to account for the different peak performance ages and development patterns of low- and high-level swimmers. Additionally, future studies should specifically analyze the annual progression rates based on the different trajectories for the various performance levels, particularly the early plateau of regional-class swimmers.

## 5. Conclusions

Female world-class finalists generally show steeper performance trajectories, while regional-class swimmers plateau earlier in their swimming career. Specialization increases for higher performance levels, as well as when swimmers approach peak performance age. World-class FR finalists show the highest degree of specialization in their main swimming stroke, potentially due to the largest spectrum of race distances. Since BU and BA swimmers can only compete in 50m, 100m and 200m races, they are less specialized. Competing in other swimming strokes allows them to increase their medal chances. Technical requirements and specialties require world-class BR finalists to have a high degree of specialization. Hence, BR was the slowest swimming stroke for BU, BA and FR swimmers, but combines well with IM. All stroke specialists also perform well in FR, potentially due to its application in the general training process for the completion of long aerobic sets. As different specialization patterns are evident for the various swimming strokes, decision makers and talent specialists should align development guidelines accordingly. Moreover, the present results aid the discussion about adjustments to the competition schedule of major championships, aiming to avoid unnecessary overlaps of commonly combined swimming strokes on the same competition day and allow swimmers to compete in races outside their main swimming stroke.

## Figures and Tables

**Table 1:** Performance progression, as indicated by swimming points, of swimmers across various performance levels, i.e., world-class finalists (850) and international- (750), national- (650) and regional-class (550) swimmers, across the various age categories.

Performance Levels	Age Categories [years]					Linear Mixed Model Analysis		
	13–14	15–16	17–18	19–20	21+			
**Butterfly**								
**850**	694 ± 34	796 ± 32 ^#^	843 ± 34	872 ± 37	879 ± 36	*R*^2^_c_ = 0.78 ICC = 0.49	(a) *F*_[4|1094]_ = 269 (b) *F*_[3|307]_ = 80 (c) *F*_[12|1098]_ = 3	*p* < 0.001 *p* < 0.001 *p* < 0.001
**750**	638 ± 54	734 ± 59 ^#^	770 ± 61	801 ± 45	822 ± 47			
**650**	590 ± 82 *	697 ± 69 *^#^	739 ± 63 *^#^	756 ± 58 *	755 ± 59 *			
**550**	554 ± 81 *	646 ± 59 *^#^	677 ± 57 *^#^	687 ± 61 *	675 ± 59 *			
**Backstroke**								
**850**	665 ± 53	765 ± 72 ^#^	817 ± 57	865 ± 56	894 ± 34	*R*^2^_c_ = 0.80 ICC = 0.57	(a) *F*_[4|1257]_ = 304 (b) *F*_[3|352]_ = 67 (c) *F*_[12|1259]_ = 3	*p* < 0.001 *p* < 0.001 *p* < 0.01
**750**	628 ± 77	736 ± 60 ^#^	776 ± 52 ^#^	797 ± 50	819 ± 52			
**650**	606 ± 80	696 ± 62 ^#^	738 ± 61 ^#^	750 ± 61 *	756 ± 57 *			
**550**	558 ± 92 *	644 ± 76 *_#_	680 ± 68 *^#^	692 ± 72 *	685 ± 68 *			
**Breaststroke**								
**850**	662 ± 85	761 ± 77 ^#^	799 ± 65	845 ± 50	887 ± 40	*R*^2^_c_ = 0.84 ICC = 0.61	(a) *F*_[4|659]_ = 413 (b) *F*_[3|185]_ = 50 (c) *F*_[12|659]_ = 2	*p* < 0.001 *p* < 0.001 *p* < 0.01
**750**	591 ± 92 *	718 ± 61 ^#^	767 ± 53 ^#^	794 ± 57	820 ± 43			
**650**	585 ± 92 *	682 ± 88 *^#^	726 ± 86 *^#^	742 ± 85 *	763 ± 65 *			
**550**	518 ± 79 *	614 ± 83 *^#^	645 ± 88 *	660 ± 80 *	673 ± 73 *			
**Freestyle**								
**850**	684 ± 67	801 ± 53 ^#^	841 ± 53	877 ± 43	891 ± 38	*R*^2^_c_ = 0.81 ICC = 0.52	(a) *F*_[4|2851]_ = 808 (b) *F*_[3|789]_ = 288 (c) *F*_[12|2855]_ = 2	*p* < 0.001 *p* < 0.001 *p* = 0.06
**750**	640 ± 80 *	738 ± 70 *^#^	784 ± 58 *^#^	807 ± 54 *^#^	816 ± 47 *			
**650**	582 ± 91 *	683 ± 72 *^#^	725 ± 63 *^#^	740 ± 60 *^#^	739 ± 57 *			
**550**	514 ± 89 *	604 ± 81 *^#^	646 ± 70 *^#^	659 ± 64 *	649 ± 58 *			
**Individual Medley**								
**850**	667 ± 84	781 ± 53 ^#^	834 ± 48	869 ± 39	890 ± 39	*R*^2^_c_ = 0.80 ICC = 0.52	(a) *F*_[4|2057]_ = 539 (b) *F*_[3|575]_ = 176 (c) *F*_[12|2059]_ = 3	*p* < 0.001 *p* < 0.001 *p* < 0.01
**750**	644 ± 83	743 ± 67 ^#^	790 ± 58 ^#^	810 ± 55	830 ± 48			
**650**	593 ± 81 *	685 ± 65 *^#^	727 ± 60 *^#^	746 ± 57 *	748 ± 57 *			
**550**	539 ± 85 *	626 ± 76 *^#^	665 ± 65 *^#^	677 ± 66 *	669 ± 65 *			

(a) Main effect: age category (13–14 vs. 15–16 vs. 17–18 vs. 19–20 vs. 21+); (b) main effect: performance level (850 vs. 750 vs. 650 vs. 550); (c) interaction effect: age category × performance level. Bonferroni post hoc comparison: * significant difference to 850 performance level (world-class finalists); # significant difference to previous age category. Abbreviations: *R*^2^_c_—*R*-squared conditional; ICC—intra-class correlation coefficient.

**Figure A1:**
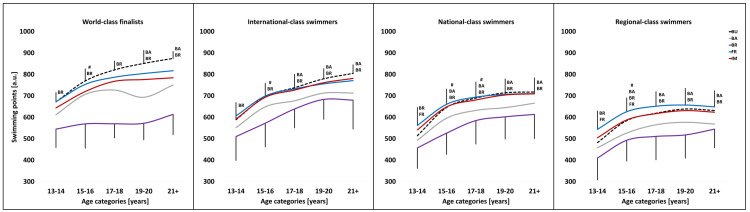
Performance progression of **butterfly** (BU) swimmers across the various age categories and performance levels. ^BA^ (backstroke), ^BR^ (breaststroke), ^FR^ (freestyle) and ^IM^ (individual medley) within the graphs indicate the significant differences between the main (dotted line) and the other swimming strokes. For the sake of clarity, significant differences to the previous age category (#) were only indicated for the main swimming stroke.

**Figure A2:**
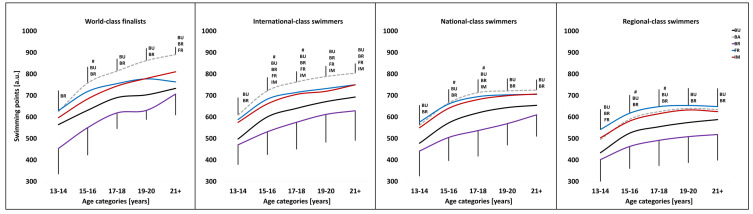
Performance progression of **backstroke** (BA) swimmers across the various age categories and performance levels. ^BU^ (butterfly), ^BR^ (breaststroke), ^FR^ (freestyle) and ^IM^ (individual medley) within the graphs indicate the significant differences between the main (dotted line) and the other swimming strokes. For the sake of clarity, significant differences to the previous age category (#) were only indicated for the main swimming stroke.

**Figure A3:**
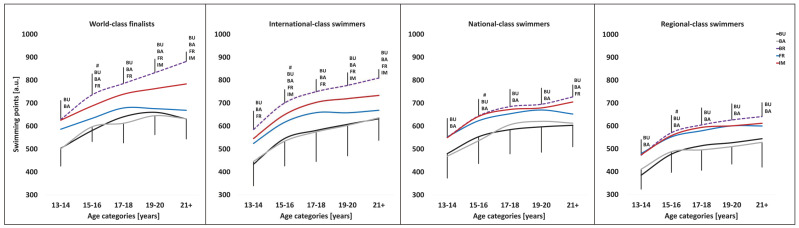
Performance progression of **breaststroke** (BR) swimmers across the various age categories and performance levels. ^BU^ (butterfly), ^BA^ (backstroke), ^FR^ (freestyle) and ^IM^ (individual medley) within the graphs indicate the significant differences between the main (dotted line) and the other swimming strokes. For the sake of clarity, significant differences to the previous age category (#) were only indicated for the main swimming stroke.

**Figure A4:**
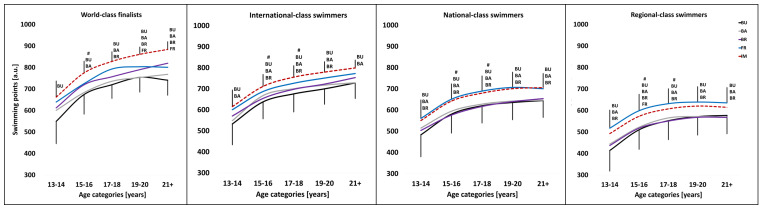
Performance progression of **individual medley** (IM) swimmers across the various age categories and performance levels. ^BU^ (butterfly), ^BA^ (backstroke), ^BR^ (breaststroke) and ^FR^ (freestyle) within the graphs indicate the significant differences between the main (dotted line) and the other swimming strokes. For the sake of clarity, significant differences to the previous age category (#) were only indicated for the main swimming stroke.

## Data Availability

All data are publicly available on Swimrankings.net.

## Supplementary Materials

The following supporting information can be downloaded at: https://www.mdpi.com/article/10.3390/jfmk10010064/s1. Table S1. Actual age at which the personal best race times occurred (mean ± SD) across all race distances within the particular swimming stroke (second step of the analysis) for world-class finalists (850), international-class (750), national-class (650) and regional-class (550) swimmers.
