# Supplementary material for: Focus on One Swimming Stroke or Compete in Multiple: How Much Specialization Is Needed to Become a World-Class Female Swimmer?
Source: J Funct Morphol Kinesiol. 2025 Feb 13;10(1):64. doi: 10.3390/jfmk10010064 (PMC11843929; doi:10.3390/jfmk10010064)
Supplement: Supplementary file 1 [file jfmk-10-00064-s001.zip › jfmk-3405209-supplementary.pdf]

Journal of
Functional Morphology and Kinesiology

MDPI

Table S1. Actual age at which the personal best race times occurred (mean ± SD) across all race distances within the particular swimming stroke (second step of the analysis) for world-class finalists (850), international-class (750), national-class (650) and regional-class (550) swimmers.

	Performance Levels			
	**850**	**750**	**650**	**550**
**Butterfly**	20.8±1.4	21.7±2.1	20.4±2.3	19.8±2.0
**Backstroke**	22.4±2.3	21.0±2.0	20.6±2.4	20.1±2.4
**Breaststroke**	23.1±2.2	22.8±2.3	21.6±2.7	21.4±2.5
**Freestyle**	21.3±2.2	21.1±2.3	20.3±2.2	20.0±2.4
**Individual Medley**	21.8±1.6	21.6±2.2	20.7±2.2	20.1±2.6
